# SET7/9-mediated methylation affects oncogenic functions of histone demethylase JMJD2A

**DOI:** 10.1172/jci.insight.164990

**Published:** 2023-10-23

**Authors:** Ruicai Gu, Tae-Dong Kim, Hoogeun Song, Yuan Sui, Sook Shin, Sangphil Oh, Ralf Janknecht

**Affiliations:** 1 Department of Cell Biology,; 2 Department of Pathology, and; 3 Stephenson Cancer Center, University of Oklahoma Health Sciences Center, Oklahoma City, Oklahoma, USA.

**Keywords:** Cell Biology, Oncology, Cancer, Molecular biology, Prostate cancer

## Abstract

The histone demethylase JMJD2A/KDM4A facilitates prostate cancer development, yet how JMJD2A function is regulated has remained elusive. Here, we demonstrate that SET7/9-mediated methylation on 6 lysine residues modulated JMJD2A. Joint mutation of these lysine residues suppressed JMJD2A’s ability to stimulate the *MMP1* matrix metallopeptidase promoter upon recruitment by the ETV1 transcription factor. Mutation of just 3 methylation sites (K505, K506, and K507) to arginine residues (3xR mutation) was sufficient to maximally reduce JMJD2A transcriptional activity and also decreased its binding to ETV1. Introduction of the 3xR mutation into DU145 prostate cancer cells reduced in vitro growth and invasion and also severely compromised tumorigenesis. Consistently, the 3xR genotype caused transcriptome changes related to cell proliferation and invasion pathways, including downregulation of *MMP1* and the *NPM3* nucleophosmin/nucleoplasmin gene. NPM3 downregulation phenocopied and its overexpression rescued, to a large degree, the 3xR mutation in DU145 cells, suggesting that NPM3 was a seminal downstream effector of methylated JMJD2A. Moreover, we found that NPM3 was overexpressed in prostate cancer and might be indicative of disease aggressiveness. SET7/9-mediated lysine methylation of JMJD2A may aggravate prostate tumorigenesis in a manner dependent on NPM3, implying that the SET7/9→JMJD2A→NPM3 axis could be targeted for therapy.

## Graphical abstract

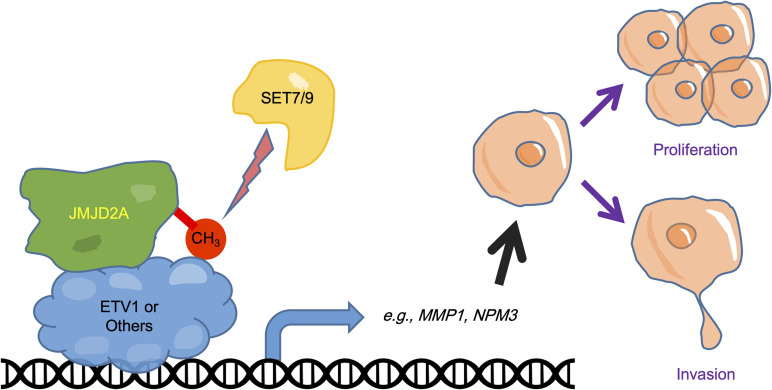

## Introduction

Prostate cancer is the most frequently diagnosed carcinoma and the second leading cause of cancer death in men in the United States (1). Sadly, metastatic disease leads to the death of the vast majority of afflicted men (2). This highlights the dire need to determine molecular events facilitating prostate cancer initiation and progression, because such knowledge could be harnessed to develop novel avenues of therapy.

Jumonji C domain–containing 2A (JMJD2A), also known as lysine demethylase 4A (KDM4A), is capable of demethylating histone H3 at tri- or dimethylated lysine 9 and 36 (3–5). Thereby, it contributes to the regulation of gene promoters and enhancers upon recruitment by various DNA-binding proteins (6, 7). JMJD2A also performs other nuclear functions by modulating DNA repair and leading to site-specific gene copy number increases (8, 9). Furthermore, JMJD2A might enhance protein synthesis in the cytoplasm (10). Hence, JMJD2A can pleiotropically affect cellular physiology.

JMJD2A interacts with and stimulates the activity of the androgen receptor, a key driver of prostate tumorigenesis, and of the ETS transcription factors ETV1 and ERG (11–13). These 2 ETS proteins are overexpressed in approximately 60% of human prostate tumors, because of chromosomal translocations (14), and initiated neoplastic transformation upon overexpression in transgenic mouse models (15–18). Downregulation of JMJD2A inhibited prostate cancer cell growth, whereas its overexpression in a transgenic mouse model led to the formation of prostatic intraepithelial neoplasia and cooperated with ETV1 to induce adenocarcinoma. Moreover, JMJD2A is overexpressed in prostate tumors, and this correlates with Gleason score and metastasis (12, 19, 20). Collectively, these data revealed that JMJD2A overexpression aids prostate tumorigenesis.

However, little is known how JMJD2A is regulated. Here, we uncovered that JMJD2A can be methylated by the SET domain–containing protein 7/9 (SET7/9; also known as SETD7). Hence, we examined how SET7/9-mediated methylation affects JMJD2A function in prostate cancer cells.

## Results

### Identification of JMJD2A as a SET7/9 substrate.

**Figure 1:**
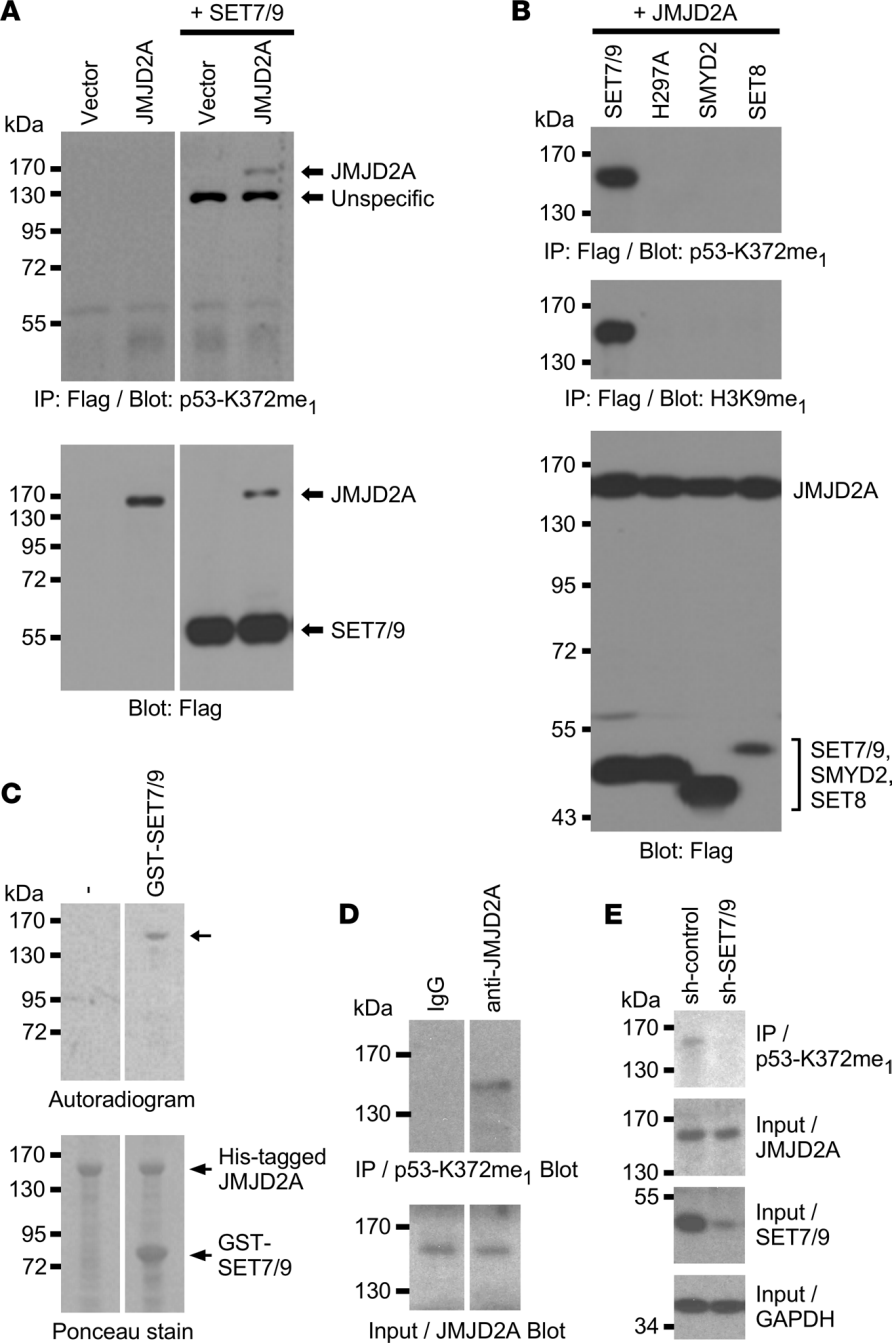
Methylation of JMJD2A by SET7/9. (**A**) Flag-tagged JMJD2A was coexpressed with Flag-tagged SET7/9 in 293T cells. After anti-Flag IP, JMJD2A methylation was assessed with Abs targeted against monomethylated K372 of p53 (top). Bottom panels show input levels for both Flag-tagged proteins, JMJD2A and SET7/9. Lanes were run on the same gel but were noncontiguous. (**B**) No methylation of Flag-JMJD2A by Flag-tagged versions of SMYD2, SET8, or the catalytically inactive H297A mutant of SET7/9 in transfected 293T cells was observed. (**C**) In vitro methylation of purified His-tagged JMJD2A by purified GST–SET7/9 in the presence of ^3^H-SAM. Lanes were run on the same gel but were noncontiguous. (**D**) Methylation of endogenous JMJD2A in DU145 cells. Cell extracts were challenged with control IgG or anti-JMJD2A Abs and respective immunoprecipitates were probed with p53-K372me_1_ Abs. Lanes were run on the same gel but were noncontiguous. (**E**) As in **D**, but in the presence of control or SET7/9 shRNA. Representative of 2 different experiments in all panels. sh, short hairpin.

JMJD2A interacts with the tumor suppressor p53 (21) that is heavily modified by lysine methylation. As such, we reasoned that p53 might be a nonhistone substrate for the JMJD2A demethylase. Hence, we examined whether JMJD2A would remove the known SET7/9-mediated monomethylation on lysine 372 of p53 (22). Although we did not find evidence for that, during these investigations we serendipitously noted that JMJD2A was recognized by an Ab raised against p53-K372me_1_ upon coexpression with SET7/9 (Figure 1A). This Ab did not recognize JMJD2A when it was coexpressed with 2 other methyltransferases, SMYD2 and SET8, or with the catalytically inactive H297A mutant of SET7/9 (Figure 1B, top). In addition, an Ab raised against H3K9me_1_ also recognized JMJD2A solely in the presence of SET7/9 (Figure 1B, middle). These data suggested that SET7/9 might directly methylate JMJD2A.

To demonstrate this, we purified recombinant JMJD2A and SET7/9 protein and performed in vitro methylation experiments with radioactive *S*-[methyl-^3^H] adenosyl-*L*-methionine (^3^H-SAM) as a methyl donor. Indeed, SET7/9 used the JMJD2A protein as a substrate for methylation (Figure 1C). In addition, we immunoprecipitated endogenous JMJD2A from human DU145 prostate cancer cells and then probed with the p53-K372me_1_ Ab (Figure 1D), revealing that endogenous JMJD2A was also methylated in DU145 cells. Likewise, JMJD2A was methylated in 3 other cell lines tested: embryonic kidney 293T, LNCaP prostate cancer and HCT116 colorectal cancer cells (Supplemental Figure 1A; supplemental material available online with this article; https://doi.org/10.1172/jci.insight.164990DS1). Notably, when we downregulated SET7/9 in DU145 cells, methylation of JMJD2A was hugely reduced (Figure 1E), lending further support to our hypothesis that JMJD2A is an in vivo substrate for SET7/9. Similarly, downregulation of SET7/9 in LNCaP prostate cancer cells reduced JMJD2A methylation, albeit to a lesser degree (Supplemental Figure 1B); this may be because residual SET7/9 in respective shRNA expressing LNCaP cells was still sufficient to methylate a large fraction of JMJD2A, or this may indicate the presence of another methyltransferase(s) in LNCaP cells capable of methylating JMJD2A.

Together, these data indicate that JMJD2A can be directly methylated by SET7/9 in vitro and in vivo. Interestingly, SET7/9 is overexpressed in prostate cancer (ref. 23 and Supplemental Figure 1, C and D), and SET7/9 protein levels positively correlate with the Gleason score (Supplemental Figure 1E), supporting the notion that enhanced posttranslational modification of JMJD2A by SET7/9 might have relevance for human prostate cancer development.

### Mapping of methylation sites.

To map the methylation sites in JMJD2A, we generated 6 different glutathione *S*-transferase (GST)–JMJD2A fusion proteins that collectively span all 1,064 aa of human JMJD2A. Of these 6 fusion proteins, only GST–JMJD2A(490–750) was methylated by SET7/9 in vitro (Supplemental Figure 2, A and B). Further subdividing JMJD2A aa 490–750 revealed that methylation occurred within aa 490–570 and 571–660, but not 661–750 (Supplemental Figure 2C).

**Figure 2:**
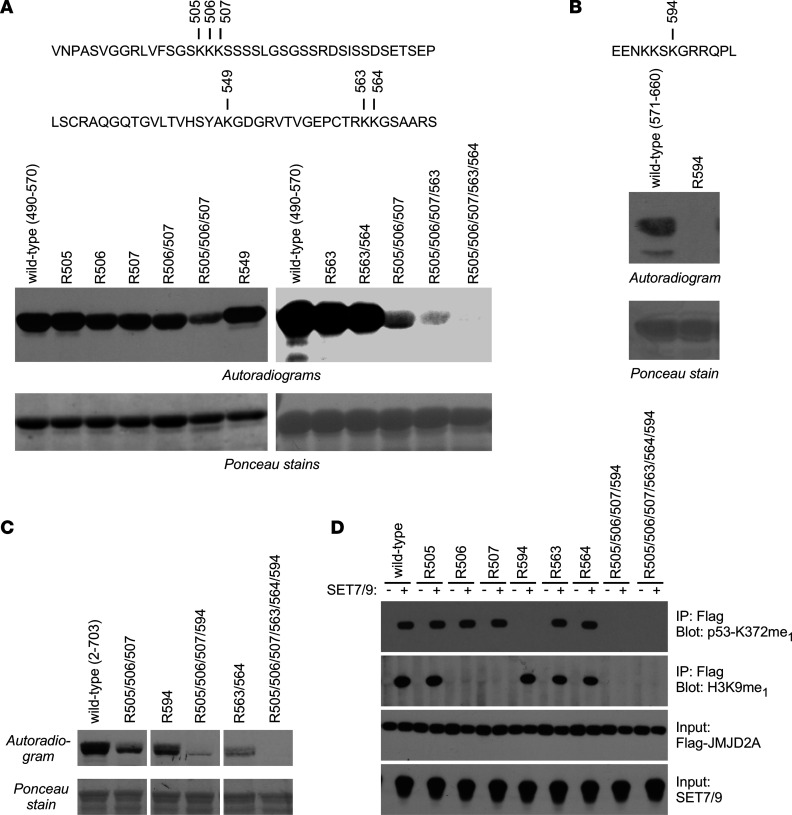
Mapping of JMJD2A methylation sites. (**A**) Indicated lysine residues within GST–MJD2A(490–570) were mutated to arginine and SET7/9-mediated in vitro methylation assessed with ^3^H-labeled SAM. The bottom panels show that comparable levels of GST–JMJD2A(490–570) fusion proteins were used, and the top panels show the JMJD2A aa sequence from 490 to 570 with all 6 lysine residues being marked. (**B**) As in **A**, but for in vitro methylation of GST–JMJD2A(571–660). The top shows JMJD2A aa 588–600. (**C**) As in **A**, but for in vitro methylation of indicated GST–JMJD2A(2–703) fusion proteins. Lanes were run on the same gel but were noncontiguous. (**D**) Flag-tagged JMJD2A (WT or indicated mutants) was expressed without or with SET7/9 in 293T cells. After anti-Flag IP, lysine methylation was assessed by blotting with 2 different methyl-specific Abs. Representative of 2 different experiments in all panels.

Within JMJD2A aa 490–570, there are 6 lysine residues (Figure 2A, top). We mutated all of them to arginine, singly or in various combinations, and then performed in vitro methylation experiments with respective GST–JMJD2A(490–570) fusion proteins. This suggested that K505 and K549 were not targeted, or were targeted only to a very low degree that was undetectable in our assay, by SET7/9, whereas mutation of K506 and/or K507 slightly reduced methylation (Figure 2A, left-side micrographs). Furthermore, the R505/506/507 mutant was less methylated than the R506/507 mutant, implying that K505 is likely also methylated by SET7/9, at least upon mutation of K506/507.

We then studied whether K563 and/or K564 were additional methyl acceptor sites. And, indeed, mutation of K563 in addition to K505/506/507 reduced methylation further, and joint mutation of K563 and K564 abolished methylation when combined with the R505/506/507 mutation (Figure 2A, right panels). This indicates that both K563 and K564 are also methylated by SET7/9.

There are also 6 lysine residues within JMJD2A aa 571–660, but only 1 fits the SET7/9 consensus methylation site (K/R) (S/T/A)K (24): namely, K594 (Figure 2B, top). Mutation of K594 to arginine completely abolished SET7/9-mediated in vitro methylation of aa 571–660 (Figure 2B), indicating that K594 is an additional SET7/9 methylation site. Together, our data strongly suggest that SET7/9 can methylate JMJD2A on up to 6 lysine residues.

We verified this with a larger GST–JMJD2A fusion protein (aa 2–703) encompassing all of those 6 lysine residues. The R505/506/507, R594, and R563/564 mutants were less methylated by SET7/9 than was the corresponding WT, and methylation was completely abolished in the R505/506/507/563/564/594 hexa-mutant (6xR; Figure 2C). We also used K→R mutants of full-length JMJD2A in 293T cells and probed for in vivo methylation by SET7/9 with methyl-specific Abs. This showed that the p53-K372me_1_ Ab pointed out methylation on K594, and the H3K9me_1_ Ab detected methylation on K506 and K507 (Figure 2D). This might be because the aa sequences surrounding K372 of p53 and K9 of histone H3 are similar to sequences flanking K594 and K506/K507 in JMJD2A, respectively (see Supplemental Figure 2D). We tried several other commercially available methyl-lysine Abs, but did not find any that could detect methylation on K505, K563, or K564. Regardless, our data indicate that JMJD2A is a substrate for the SET7/9 lysine methyltransferase and thus is potentially regulated by a corresponding posttranslational modification.

### Impact of methylation on transactivation.

**Figure 3:**
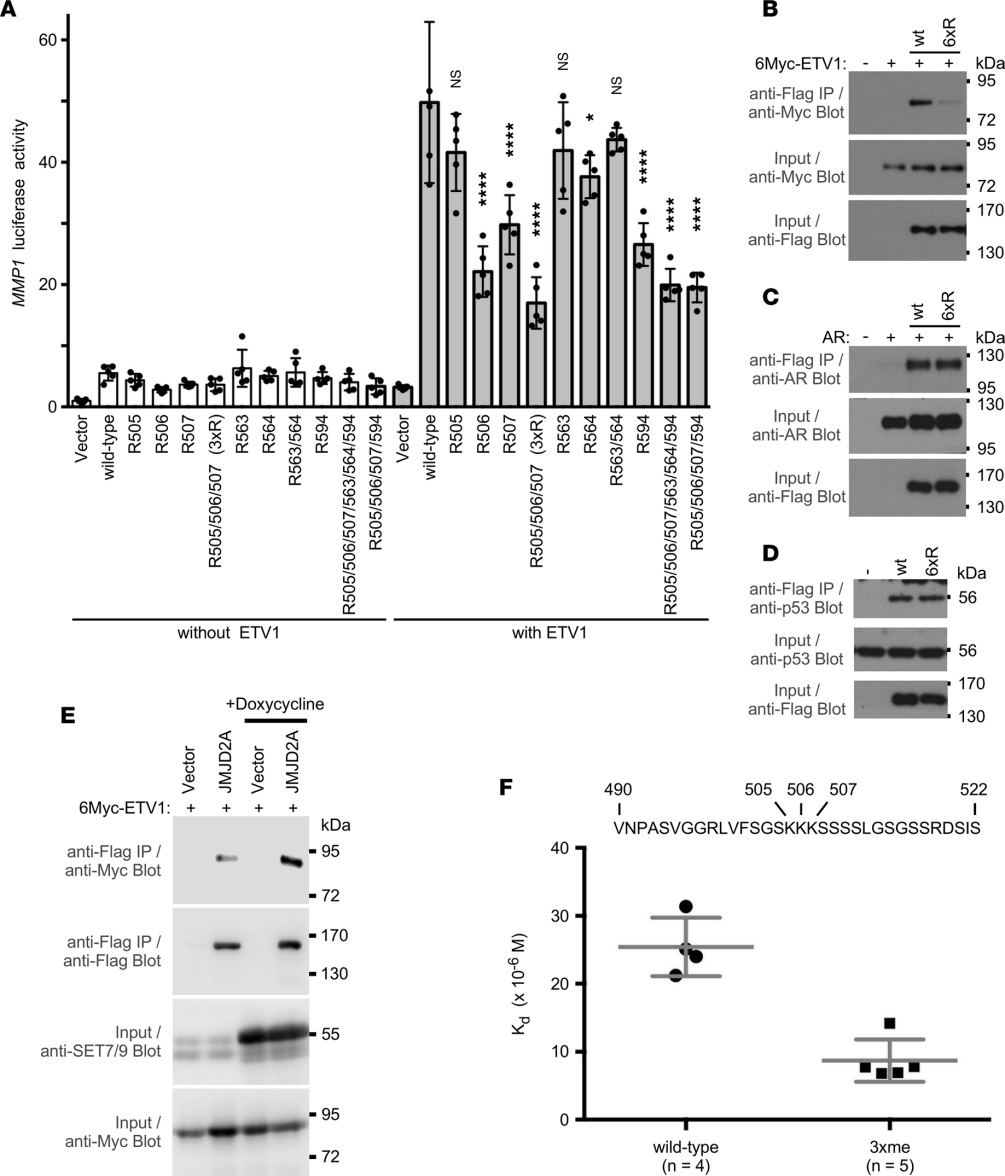
Methylation of JMJD2A affects cooperation with ETV1. (**A**) LNCaP prostate cancer cells were transfected with an *MMP1* LUC reporter plasmid and Flag-tagged JMJD2A (the WT or indicated mutants thereof) in the absence or presence of an ETV1 expression plasmid. Shown are averages with SD (*n* = 5). Statistical significance was assessed with 1-way ANOVA (Dunnett’s multiple comparison test) and comparison was to WT JMJD2A in the presence of ETV1. *P < 0.05, ****P < 0.0001. (**B**) 6Myc-tagged ETV1 was coexpressed with either WT Flag-JMJD2A or its 6xR (R505/506/507/563/564/594) mutant in 293T cells. After anti-Flag IP, coprecipitated ETV1 was detected by anti-Myc Western blotting. The bottom 2 panels show input levels for 6Myc-ETV1 and Flag-JMJD2A proteins. (**C**) Likewise, interaction of JMJD2A with ectopically expressed androgen receptor (AR) in the presence of 50 nM dihydrotestosterone. (**D**) Analogous JMJD2A complex formation with endogenous p53. (**E**) As in **B**, interaction of Flag-tagged JMJD2A with Myc-tagged ETV1 was assessed by co-IP. Addition of doxycycline (0.3 μg/mL) resulted in overexpression of SET7/9. (**F**) Binding of unmethylated (WT) or triple methylated (on K505, K506, and K507; 3xme) peptide spanning JMJD2A aa 490–522 to the GST–ETV1(249–477) fusion protein; unpaired, 2-tailed *t* test (*P* = 0.0003). (**B**–**E**) Representative of at least 2 different experiments.

To determine if methylation affects the transactivation potential of JMJD2A, we performed reporter gene assays with the *MMP1* promoter that is jointly stimulated by JMJD2A and its interaction partner, the DNA-binding transcription factor ETV1 (12, 25). In the absence of overexpressed ETV1, ectopic JMJD2A slightly activated the *MMP1* LUC reporter construct in human LNCaP prostate cancer cells, but there was no statistically significant difference between WT JMJD2A and the various methylation site mutants (Figure 3A). As expected, ectopic ETV1 greatly synergized with WT JMJD2A in stimulating the *MMP1* promoter. Mutation of K506, K507, K564, or K594 alone significantly reduced this synergy, and various combination mutants were even less efficacious. Notably, the R505/506/507 (3xR) triple mutant was as much deactivated as the R505/506/507/563/564/594 hexa-mutant, implying that, in particular, methylation on K505, K506, and K507 is relevant for stimulating the transactivation function of JMJD2A (Figure 3A).

One way methylation of JMJD2A may further ETV1-dependent transcription would be through enhancing JMJD2A-ETV1 complex formation. Notably, all of the 6 identified methylation sites are encompassed within JMJD2A aa 490–750 that were previously shown to mediate JMJD2A’s interaction with ETV1 (12). Indeed, we observed that the R505/506/507/563/564/594 hexa-mutant bound less avidly to ETV1 compared with WT JMJD2A (Figure 3B). We also observed that mutation of K505/506/507 was as effective as mutation of all 6 methylation sites in lessening the interaction between JMJD2A and ETV1, whereas mutation of K563/564 or K594 had no effect (Supplemental Figure 3A). We also tested whether methylation would affect the known (11, 21) binding of JMJD2A to the androgen receptor or p53. However, mutation of the 6 methylation sites in JMJD2A had no impact on the interaction with those 2 proteins (Figure 3, C and D).

To further support the notion that methylation of JMJD2A enhances its affinity toward ETV1, we overexpressed SET7/9 and assessed how this would affect the complex formation between JMJD2A and ETV1. We used a doxycycline-inducible SET7/9 expression construct. As expected, we observed that more ETV1 coimmunoprecipitated with JMJD2A in the presence of doxycycline (Figure 3E). Moreover, we directly measured the affinity of a peptide encompassing JMJD2A aa 490–522 toward a fusion between GST and the C-terminal half of ETV1 (aa 249–477) that was previously shown to facilitate binding to JMJD2A (12). This was done with microscale thermophoresis (MST). We found that the K_d_ of the peptide became approximately 3-fold reduced (from 25.4 μM to 8.7 μM) when the peptide was methylated on K505, K506, and K507 (Figure 3F and Supplemental Figure 3B). This corroborates that methylation on these 3 lysine residues enhances binding of JMJD2A to ETV1, providing 1 potential mechanism by which methylation of K505, K506, and K507 can modulate the function of JMJD2A.

### Methylation of JMJD2A promotes its oncogenic properties.

**Figure 4:**
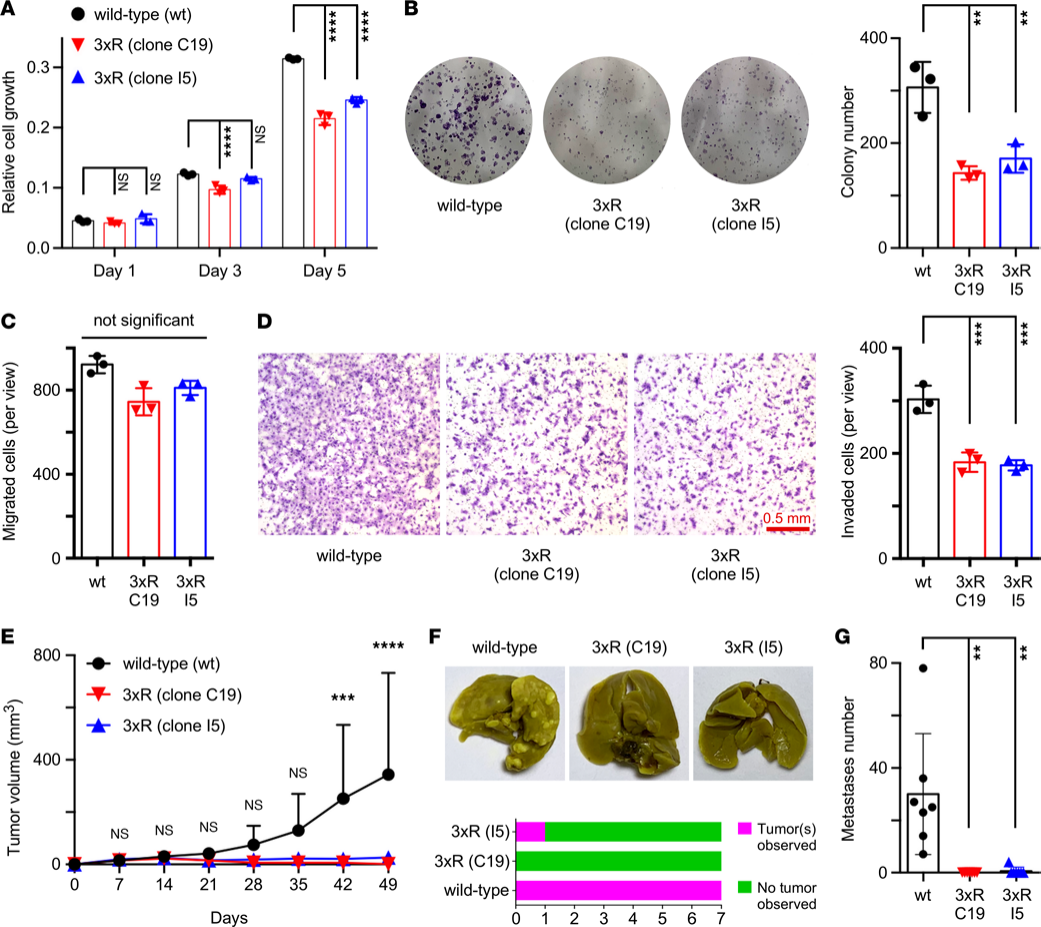
3xR mutation inhibits JMJD2A function in DU145 prostate cancer cells. Assessment of (**A**) cell growth, (**B**) clonogenic activity, (**C**) migration, and (**D**) invasion. Shown are representative images and averages with SD determined with 2-way (**A**) or 1-way (**B**–**D**) ANOVA (Tukey’s multiple comparisons test; *n* = 3). (**E**) Tumor volume after s.c. injection into nude mice; 2-way ANOVA (Holm-Šídák multiple comparisons test; *n* = 8). (**F**) Representative photographs of lungs after tail vein injection into nude mice and χ^2^ contingency test (*n* = 7; *P* = 0.0002). (**G**) Corresponding number of lung metastases observed; 1-way ANOVA (Tukey’s multiple comparisons test; *n* = 7). **P < 0.01, ***P < 0.001, ****P < 0.0001.

To determine how methylation affects the ability of JMJD2A to influence prostate cancer cells, we elected to replace lysine by arginine residues through CRISPR/Cas9-mediated homologous recombination. Because methylation on K505, K506, and K507 appeared to be most critical for JMJD2A function, we simultaneously replaced these 3 lysines with arginine residues in human DU145 prostate cancer cells (Supplemental Figure 4A). We obtained 2 respective, independent 3xR clones, C19 and I5, in which JMJD2A was expectedly no longer recognized by the H3K9me_1_ Ab (Supplemental Figure 4B). Both clones displayed diminished cell and clonogenic growth as well as invasion, compared with WT DU145 cells. Migration was not statistically significantly altered (Figure 4, A–D), and neither was JMJD2A protein stability (Supplemental Figure 4, C and D). Similarly, overexpression of JMJD2A in LNCaP prostate cancer cells revealed that the 3xR and the 6xR mutations suppressed the ability to invade but not to migrate (Supplemental Figure 5). In addition, although overexpression of WT JMJD2A had no impact on LNCaP cell growth, the 3xR and 6xR mutants acted in a dominant-negative fashion by slightly, yet significantly, reducing cell growth (Supplemental Figure 5B). Please note that the 3xR mutant was as efficient as the 6xR mutant in altering LNCaP cell growth and invasion, emphasizing again that methylation on K505, K506, and K507 essentially determines the biological consequences of SET7/9-mediated JMJD2A posttranslational modification.

Moreover, we subcutaneously injected DU145 cells into nude mice and observed a nearly total block of tumor growth with both the C19 and I5 3xR clones (Figure 4E). We also injected cells via the tail vein and monitored the establishment of lung tumors. Only 1 of the 14 mice injected with a 3xR clone developed lung tumors, compared with 7 of 7 for WT cells (Figure 4F). Also, there were 4 observable lung tumors with the sole positive 3xR specimen, which contrasted with a range of 7–78 lung tumors per mouse for WT cells (Figure 4G). Together, our in vitro and in vivo data implicate methylation of JMJD2A as needed for its oncogenic potential.

### Global transcriptomic changes in 3xR DU145 cells.

**Figure 5:**
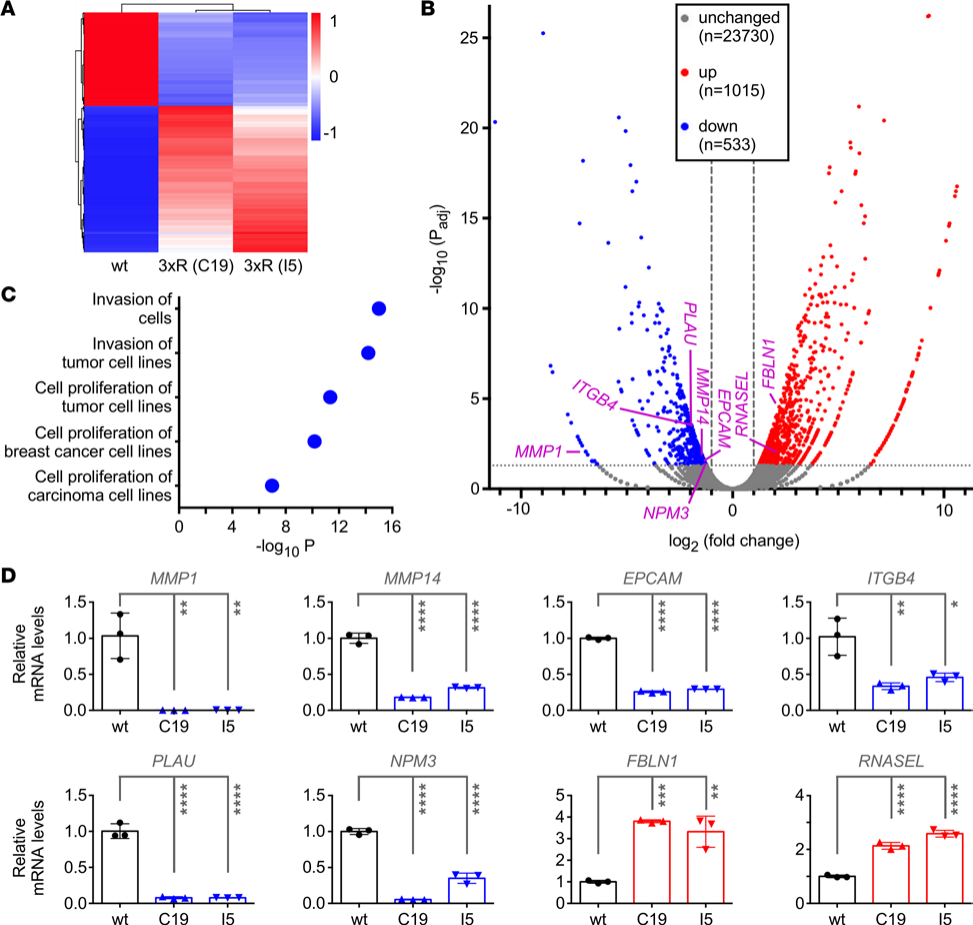
Transcriptome differences between WT and JMJD2A-3xR DU145 cells. (**A**) Heatmap with differentially expressed genes. (**B**) Volcano plot showing the average adjusted *P* value (*P*_adj_) and fold-change values for wt versus 3xR C19 and wt versus 3xR I5. Only genes with *P*_adj_ < 0.05 and |fold change| > 2 were considered to be differentially regulated. (**C**) Ingenuity pathway analysis showing the 5 hits with the lowest *P*_adj_ and |*z* score| > 1. (**D**) Quantitative RT-PCR validation of differential expression of indicated genes between WT DU145 cells and the 2 3xR clones, C19 and I5. Shown are relative mRNA levels (normalized to *GAPDH*); 1-way ANOVA (Tukey’s multiple comparisons test; *n* = 3). *P < 0.05, **P < 0.01, ***P < 0.001, ****P < 0.0001.

To gain mechanistic insights into how the 3xR mutation affects JMJD2A function in DU145 cells, we performed RNA-Seq. Approximately 1,500 differentially expressed genes were detected when comparing WT with 3xR cells (Figure 5, A and B). Ingenuity pathway analysis showed that pathways related to cell invasion and proliferation were downregulated in the 3xR cells (Figure 5C). We then selected differentially expressed genes known to promote (*MMP1*, *MMP14*, *EPCAM*, *ITGB4*, *PLAU*) or inhibit (*FBLN1*, *RNASEL*) prostate cancer cell invasion and/or proliferation (see Discussion) and validated the RNA-Seq data by RT-PCR for these genes (compare Figure 5B with Figure 5D). Of note, SET7/9 ablation only partially mimicked the 3xR mutation by leading to the downregulation of *MMP1*, *ITGB4*, and *PLAU* but not of *MMP14* and *EPCAM* mRNA, and also did not elicit statistically significant *FBLN1* or *RNASEL* upregulation (Supplemental Figure 6), implying that SET7/9 does not exclusively act through the methylation of JMJD2A.

Notably, 1 of the most downregulated genes in 3xR cells was *MMP1* (see Figure 5, B and D). MMP1 overexpression has been reported in prostate cancer (26), which we corroborated through IHC analyses (Supplemental Figure 7A). Downregulation of MMP1 with 2 independent shRNAs in DU145 cells had no significant impact on cell growth but greatly diminished invasion (Supplemental Figure 7, B–D). The latter is consistent with previous studies showing that DU145 cell invasion was reduced upon treatment with an MMP1 inhibitor (27, 28). These results indicate that reduction of *MMP1* transcription in 3xR cells may contribute to their reduced invasion capacity, but the results cannot explain their compromised growth.

### NPM3 as a novel prostate cancer promoter.

**Figure 6:**
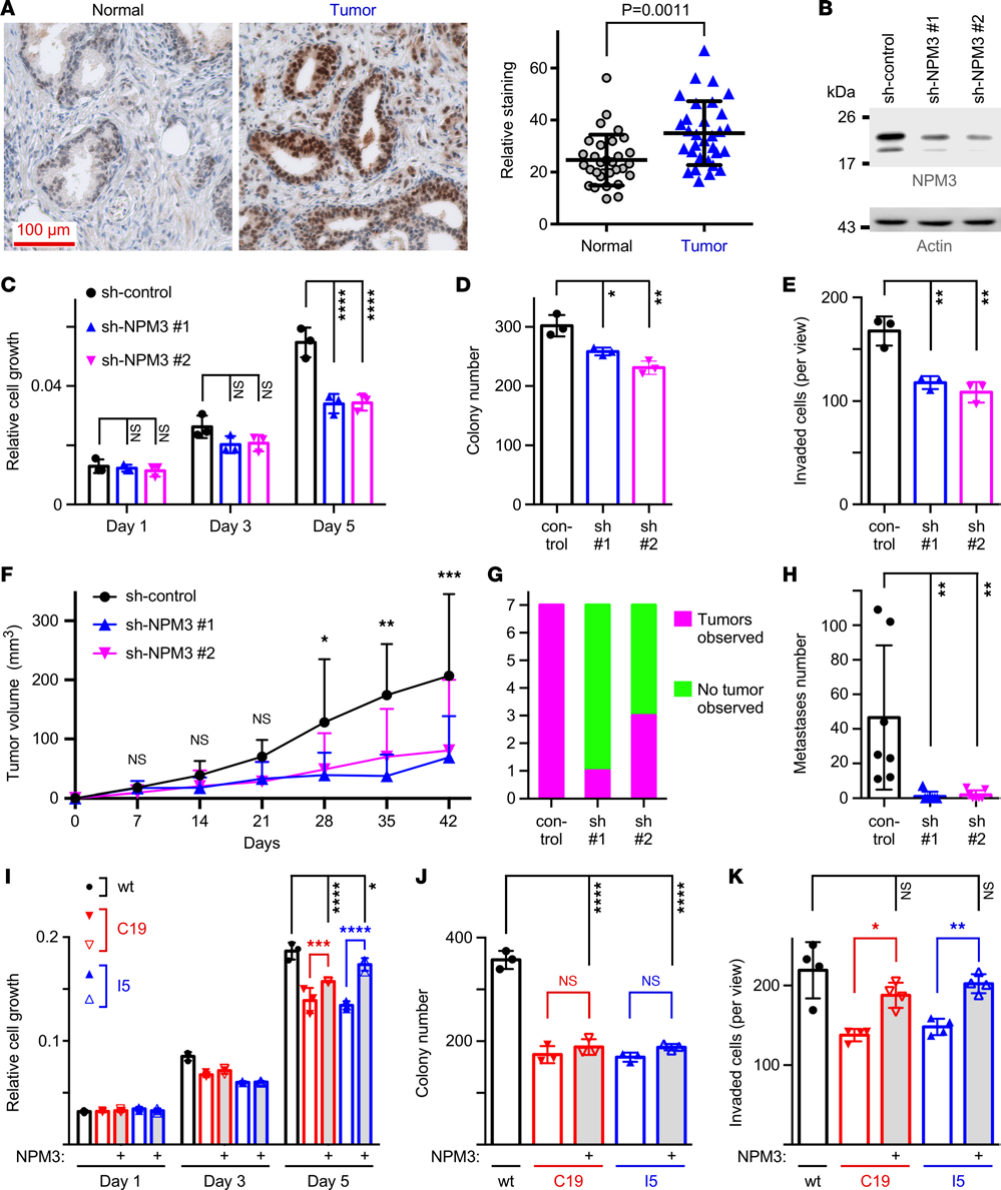
Oncogenic properties of NPM3. (**A**) Example of NPM3 immunoreactivity in normal and cancerous prostate tissue and quantitation of all 31 matched normal and tumor samples (paired, 2-tailed *t* test). (**B**) Western blots showing downregulation of NPM3 with 2 independent miRNA-based shRNAs in DU145 cells. (**C**) Cell growth, (**D**) clonogenic activity, and (**E**) invasion were then assessed. Shown are averages with SD; 2-way (**C**) or 1-way (**D** and **E**) ANOVA (Tukey’s multiple comparisons test; *n* = 3). (**F**) Tumor volume after s.c. injection into nude mice; 2-way ANOVA (Tukey’s multiple comparisons test; *n* = 7). (**G**) Lung tumor incidence after tail vein injection; χ^2^ contingency test (*n* = 7; *P* = 0.0048). (**H**) Corresponding number of lung metastases observed; 1-way ANOVA (Dunnett’s multiple comparisons test; *n* = 7). (**I**) Changes in growth (2-way ANOVA with Tukey’s multiple comparisons test; *n* = 3), (**J**) clonogenic activity (1-way ANOVA with Tukey’s multiple comparisons test; *n* = 3), or (**K**) invasion (1-way ANOVA with Tukey’s multiple comparisons test; *n* = 4) upon NPM3 overexpression in 3xR cells. *P < 0.05, **P < 0.01, ***P < 0.001, ****P < 0.0001.

To find effectors of methylated JMJD2A needed for stimulation of cell growth, we then focused on other differentially expressed genes whose relevance for prostate cancer has been unknown. From these genes, we selected for deeper study nucleophosmin/nucleoplasmin 3 (*NPM3*), which was downregulated in 3xR cells (see Figure 5, B and D) and also upon SET7/9 ablation (see Supplemental Figure 6A). Bioinformatics revealed that *NPM3* mRNA is upregulated in prostate tumors and even more so in metastases (Supplemental Figure 8, A–E). We corroborated that NPM3 is also overexpressed at the protein level in prostate tumors (Figure 6A). In addition, high *NPM3* levels were associated with a higher Gleason score, more disease recurrence, and lower overall survival (Supplemental Figure 8, F–H). Notably, NPM3 downregulation in DU145 cells largely phenocopied the effects observed with the JMJD2A-3xR mutant by eliciting a significant reduction of cell growth, clonogenic activity, and invasion (Figure 6, B–E). Xenograft analyses demonstrated that NPM3 downregulation also resulted in less tumorigenesis (Figure 6, F–H). Furthermore, NPM3 overexpression in JMJD2A–3xR DU145 cells significantly enhanced cell growth and invasion but not clonogenic activity (Figure 6, I–K; see Supplemental Figure 8I for respective Western blots showing NPM3 protein levels), indicating that NPM3 can considerably, but not completely, rescue the deleterious consequences of the 3xR mutation. Together, these results nominate NPM3 as a seminal downstream effector of methylated JMJD2A.

## Discussion

In this report, we have uncovered that JMJD2A is a substrate for the SET7/9 methyltransferase and identified 6 lysine residues as potential methylation sites. Furthermore, we showed that abrogation of JMJD2A methylation severely compromised its ability to stimulate DU145 prostate cancer cell growth and invasion in vitro as well as tumor formation in vivo. Finally, we identified NPM3 as a downstream effector of JMJD2A and revealed that NPM3, in its own right, can promote prostate tumorigenesis.

Although SET7/9 was originally identified as an H3K4 methyltransferase, methylation of nonhistone proteins is likely more relevant because SET7/9 was incapable of methylating histone H3 within nucleosomes and global H3K4 methylation was unaffected in SET7/9 KO cells (22, 29, 30). Several SET7/9 client proteins were identified, and the role of SET7/9-mediated methylation in tumorigenesis appears to be context dependent (31). For instance, SET7/9 has been shown to methylate and thereby compromise the function of the coactivators and oncoproteins, YAP1 and β-catenin, and, in that way, potentially suppressing tumorigenesis (32, 33). On the other hand, methylation of the androgen receptor augmented its transcriptional functions and, accordingly, SET7/9 supported growth and survival of androgen-dependent prostate cancer cells (23, 34). Likewise, our results indicate that methylation of JMJD2A by SET7/9 stimulates the transcriptional activity of JMJD2A and its ability to promote prostate cancer cell growth and metastasis. In DU145, but not LNCaP, cells, SET7/9 ablation greatly reduced JMJD2A methylation, indicating that JMJD2A methylation is essentially solely governed by SET7/9 activity in DU145 cells. However, this may not be the case in LNCaP cells. For instance, SET1 or MLL methyltransferases, which, like SET7/9, methylate H3K4 and may thus have a similar substrate specificity (35), might also induce JMJD2A methylation in LNCaP cells. Another difference between LNCaP and DU145 cells is that only the former have detectable levels of endogenous ETV1 (see Supplemental Figure 9). This suggests that our finding that methylation of JMJD2A enhances its binding to ETV1 is irrelevant for the observed effects of JMJD2A methylation in DU145 cells, but it may account for the anti-oncogenic effects of the 3xR and 6xR JMJD2A mutants in LNCaP cells. At present, we do not know if and what other DNA-binding transcription factor(s) in DU145 cells might preferentially form complexes with methylated JMJD2A, thereby potentially effecting the oncogenic consequences of SET7/9-mediated methylation of JMJD2A.

KO of *Set7/9* in mice did not cause any obvious developmental defects or change of life span (36, 37), suggesting that SET7/9 inhibitors will have little impact on normal cells. This is desirable for treating cancer cells with SET7/9 inhibitors. The fact that maximal androgen receptor function is dependent on SET7/9-mediated methylation (23, 34) already suggested that SET7/9 inhibitors are potentially beneficial for therapy of androgen-dependent prostate cancer. Because mutation of JMJD2A methylation sites in the androgen-independent DU145 cells severely compromised their oncogenicity, the present study strongly suggests an additional potential utility of SET7/9 inhibitors for castration-resistant prostate cancer. Selective small-molecule SET7/9 inhibitors have already been described (38, 39) that could be tested for prostate cancer therapy.

Previous reports using pharmacological or genetic approaches to inhibit JMJD2A clearly highlighted that inhibition of JMJD2A enzymatic activity by small-molecule drugs may be effective in treating prostate cancer (12, 19, 20). However, this is hampered by the fact that the catalytic center of JMJD2A is highly homologous to those of JMJD2B and JMJD2C (6), accounting for the fact that no specific inhibitor for just JMJD2A has yet been reported. Although *Jmjd2a*-KO mice are viable and display no obvious pathological phenotype, combined *Jmjd2a* and *Jmjd2c* KO resulted in embryonic lethality and, in particular, impaired stem cell self-renewal (40), implying that a pan-JMJD2 inhibitor would not be well tolerated in humans. In contrast, the K505, K506, and K507 methylation sites of JMJD2A are not conserved in JMJD2B and JMJD2C (see Supplemental Figure 10), suggesting that peptide mimetics of these JMJD2A methylation sites could specifically inhibit JMJD2A methylation, and thereby its oncogenic function, without serious adverse effects.

One JMJD2A downstream effector identified in our RNA-Seq experiments is NPM3. It and its homologs, NPM1 and NPM2, may function as histone chaperones. NPM3 forms oligomers especially with NPM1, and both of these proteins are preferentially localized within nucleoli (41–43), but they may also synergize in transcription activation outside of the nucleoli (44). NPM3 has been reported to inhibit ribosome biogenesis (41), which ought to inhibit cell proliferation, but NPM3 apparently also increased embryonic stem cell growth (42). This suggests that NPM3 may exert both pro- and antiproliferative effects, which may be context dependent, and is similar to what has been observed for NPM1 (45). Our data show that NPM3 is overexpressed in prostate tumors and marks aggressive disease, suggesting that NPM3 drives prostate tumorigenesis. Consistently, NPM3 downregulation inhibited DU145 cells both in vitro and in vivo, leading to reduced tumor formation at the primary injection site as well as in the lung.

To our knowledge, this is the first time that NPM3 has been shown to stimulate tumorigenesis in vivo and, therefore, the present study uncovered NPM3 as a potential oncoprotein. While this study was under revision, another report demonstrated that NPM3 can stimulate proliferation and migration of lung adenocarcinoma cells in vitro (46). Accordingly, NPM3 inhibitors might be useful for prostate and lung cancer therapy. Although such inhibitors have not been developed yet, they have in the case of NPM1. The disruption of NPM1 oligomerization by NSC348884 has been shown to lead to apoptosis in cancer cells (47–49). Whether NSC348884 also disrupts oligomers between NPM3 and NPM1 and thereby additionally inhibits NPM3 function is unknown, but disruption of NPM3 oligomerization appears to be a plausible approach for disabling its function.

NPM3 overexpression did not fully rescue all the defects elicited upon mutation of JMJD2A methylation sites, indicating that there is not just 1 downstream effector that mediates the oncogenic effects of methylated JMJD2A. Indeed, our RNA-Seq data uncovered several other potential downstream effectors of JMJD2A. This included 2 matrix metallopeptidases, MMP1 and MMP14, both of which are overexpressed in human prostate tumors and promote invasion and metastasis of prostate cancer cells (26, 27, 50, 51). Although inhibition of matrix metallopeptidases, including MMP1, is, in principle, attractive for cancer therapy, the complexities of their functions as well as the nonselectivity of current inhibitors are likely underlying causes for their failure in clinical trials (52). Another proteolytic enzyme, PLAU, was downregulated in 3xR cells, and it is another activator of prostate cancer cell invasion and metastasis (50, 53). Furthermore, EPCAM, which mediates cell adhesion, and ITGB4, an integrin that can regulate cellular growth and movement, were downregulated in 3xR cells, and both have been shown to be overexpressed in prostate cancer and facilitate metastasis (53–57). On the other hand, *FBLN1*, which encodes an extracellular glycoprotein, was upregulated in 3xR cells, suggesting that FBLN1 may suppress tumorigenesis. Consistently, *FBLN1* downregulation has been observed in human prostate cancer, which may lead to reduced cell death and thereby promote tumorigenesis (58–60). Another gene found upregulated in 3xR cells was *RNASEL*. Inactivating mutations in the encoded RNase have been associated with increased prostate cancer risk (61–64). Dovetailing with this, RNASEL ablation in prostate cancer cells promoted motility as well as tumor growth and metastasis (65, 66). These examples illustrate how mutation of JMJD2A methylation sites can pleiotropically affect the transcriptome of prostate cancer cells and thereby their oncogenic potential.

Together, this study elucidated how JMJD2A can be regulated by SET7/9-mediated methylation and nominated the SET7/9→JMJD2A→NPM3 axis as a target for the treatment of prostate tumors. Because JMJD2A performs oncogenic functions not only in prostate tissue (6, 7), the conclusions of this study are likely to pertain to many other malignancies beyond prostate cancer.

## Methods

### Cell lines.

The following cell lines were obtained from the American Type Culture Collection: LNCaP (catalog CRL-1740), DU145 (catalog HTB-81), PC-3 (catalog CRL-1435), RWPE-1 (catalog CRL-3607), 293T (catalog CRL-3216), and HCT116 (catalog CCL-247). Modified DU145 cells were obtained from Synthego. Cell lines were immediately amplified upon receipt and aliquots were frozen down. After approximately 1–3 months of growth, cells were discarded and replaced with a fresh aliquot. Approximately every other month, cells were monitored for *Mycoplasma* contamination.

### IPs, Western blotting, and IHC.

Human embryonic kidney 293T cells were transfected by the calcium phosphate coprecipitation method with expression vectors for indicated proteins (67). Cells were lysed 36 hours after transfection and IPs performed essentially as described previously (68). Precipitated proteins were then resolved on SDS-PAGE, followed by transfer to PVDF membrane and detection with appropriate Abs (69). IHC was performed as described before (70). All Abs we used are listed in Supplemental Table 1.

### In vitro methylation.

Fusions of JMJD2A aa or SET7/9 with GST were produced in *E*. *coli* and affinity purified with the help of glutathione agarose beads (71). The 6His/Flag-tagged JMJD2A was expressed with the help of a baculovirus expression system and purified on Ni^2+^-NTA agarose (13). These recombinant proteins (1–3 μg) were incubated for 2 hours at 30°C in the presence of 1 μM ^3^H-SAM (60 Ci/mmol) in 50 mM Tris-HCl (pH 8.5), 5 mM MgCl_2_, and 4 mM DTT. Samples were then subjected to SDS-PAGE, transferred to PVDF membrane, and visualized with Ponceau S staining (72). The dried membrane was sprayed with EN^3^HANCE (PerkinElmer) 4 times, waiting for 10 minutes between each application. After a final drying for 30 minutes, membranes were exposed to film at –80°C without intensifying screen.

### LUC reporter gene assay.

Human LNCaP prostate cancer cells were grown in poly-l-lysine–coated 6-well plates (73). Then, cells were transfected with a mixture of 1 μg of pBluescript KS^+^, 1 μg of pGL2-*MMP1*(–525/+15) LUC reporter plasmid (25), 100 ng of empty vector pEV3S or Flag-tagged JMJD2A expression vector, and 5 ng of pEV3S or CMV-ETV1 expression plasmid using 8 μg of polyethylenimine. After 8 hours, cells were washed once with 2 mL of PBS and incubated with 2 mL or growth media for another 40 hours. Finally, cells were lysed in 350 μL of 25 mM Tris (pH 7.8), 2 mM EDTA, 2 mM DTT, 10% glycerol, and 1% Triton X-100 (74), and LUC activities were determined as described (75).

### MST.

Peptides spanning JMJD2A aa 490–522, either unmethylated or monomethylated at K505, K506, and K507, were synthesized by GenScript with C-terminal amidation. GST–ETV1(249–477) fusion protein was produced in *E*. *coli* and purified as described (76). Measurement of binding of peptides to the GST–ETV1(249–477) fusion protein was performed through MST in a Monolith NT.115 apparatus (NanoTemper Technologies) according to the manufacturer’s recommendations. The binding buffer was PBS supplemented with 0.05% Tween-20. The concentration of GST–ETV1(249–477) was 300 nM, the concentration of an anti-GST mouse mAb coupled to the Alexa Fluor 647 dye (MA4-004-A647; Invitrogen) was 20 nM, and 15 serial dilutions of peptide (starting concentration: 50 μM) were used. Data analysis was done with Affinity Analysis (MST), version 2.3, software (NanoTemper Technologies).

### RNA-Seq and RT-PCR.

RNA was prepared as described (77). Then, mRNA-Seq was performed at Novogene with a 150 bp paired-end sequencing strategy and *at least* 20 million read pairs per sample. Differential expression analysis was performed with the edgeR software package (version 3.22.5) and the *P* values were adjusted using the Benjamini-Hochberg method. A corrected *P* value of less than 0.05 and an absolute fold-change of at least 2 were set as the threshold for significantly differential gene expression. For RT-PCR, RNA was reverse-transcribed with the GoScript Reverse Transcription System (catalog A5004; Promega) using p(dN)_6_ primers and then amplified by PCR according to standard procedures (78). Relative RNA expression was determined with the comparative ΔCt method and normalized to *GAPDH* levels as described elsewhere (79). Primer sequences are listed in Supplemental Table 2.

### Cell growth and clonogenic assays.

A total of 2,500 cells were seeded into 96-well plates and grown for indicated times, after which growth was assayed with the PrestoBlue cell viability kit (Invitrogen) (77). For clonogenic assays, 2,500 cells were seeded into 6-well plates and colony formation revealed by staining with crystal violet after 14 days (80).

### Migration and invasion assays.

Cells were treated with 10 μg/mL mitomycin C for 2 hours and then split with trypsin/EDTA. A total of 5 × 10^4^ cells were seeded on top of 8 μm chambers without (cell culture inserts; catalog 10769-242, VWR) and with Matrigel (BioCoat Growth Factor Reduced Matrigel Invasion Chambers; catalog 354483, Corning) and then grown with 0.1% FBS-containing medium on top and 10% FBS-containing medium on the bottom (81). After 48 hours, cells were removed from the top of the filter inserts, and cells that had passed through the 8 μm pore membranes were fixed with methanol and revealed with the Harleco Hemacolor Stain Set (MilliporeSigma). Images were captured by microscopy and quantitation was performed by counting cells in 3 random fields at ×10 original magnification.

### Xenografts.

Male, 5-week-old nude mice (*Fox1^nu^/Fox1^nu^*; catalog 007850) were purchased from Jackson Laboratory and acclimated for 2 weeks before use. DU145 cells of 95% viability were injected s.c. into the right flank (82). A total volume of 200 μL, consisting of 2 × 10^6^ cells resuspended in 100 μL PBS and 100 μL growth-factor–reduced Matrigel (catalog 354230, Corning), was injected using a 27G needle. Tumor size was measured weekly with a caliper and tumor volume was calculated by the formula width × width × length/2. For tail vein injections, 2.5 × 10^6^ cells were resuspended in 200 μL of PBS and injected through a 27G needle. Mice were euthanized 70 days after injection, and lung tissue was collected and fixed in Bouin’s solution for 24 hours. After twice washing with 75% ethanol, micrographs were taken and macroscopically visible metastases were counted.

### RNA interference and viral transduction.

shRNA sequences, 21 nucleotides long, targeting *NPM3* were embedded within the *miR30* gene and cloned into a lentiviral vector (83); *NPM3* cDNA was cloned into retroviral vector pQCXIH. Virus was produced in 293T cells as described (84) and used to infect DU145 cells 3 times over 12 hours. Stably transduced cells were selected with 2 μg/mL puromycin or 200 μg/mL hygromycin B for 4 days, after which cells were split for use in the indicated assays. The sequences targeted within *NPM3* mRNA were 5′-CCUGACAAGUUUCAACAAUUG-3′ (sh-NPM3 1) and 5′-CCUGUGAGAAUUGGAGGUUAG-3′ (sh-NPM3 2). For downregulation of SET7/9, shRNA was cloned into pSIREN-RetroQ and respective retrovirus produced and used for DU145 cell infection as described above. The sequences targeted within *SET7/9* mRNA were 5′-GCACCUGGACGAUGACGGAUUAC-3′ (sh-SET7/9 1) and 5′-GGAGAUGACUGGAGAGAAG-3′ (sh-SET7/9 2).

### Statistics.

The number of replicates is indicated within each figure legend, as is the statistical test applied. If not otherwise indicated, shown are averages with SD. Statistical analysis was performed with GraphPad Prism, version 6.0h. Statistical significance was assumed when *P* < 0.05.

### Study approval.

All mouse experiments were approved by the University of Oklahoma Health Sciences Center IACUC.

### Data availability.

RNA-Seq data have been deposited with the National Center for Biotechnology Information under BioProject ID PRJNA753498. Other data are available in the Supporting Data Values file.

## Author contributions

RG, TDK, HS, and RJ designed and performed experiments. RG, TDK, HS, YS, SS, SO, and RJ analyzed and interpreted data. RJ supervised this study.

## Supplementary Material

Supplemental data

Supporting data values

## Version 1 10/23/2023

Electronic publication
