# Supplementary material for: SET7/9-mediated methylation affects oncogenic functions of histone demethylase JMJD2A
Source: JCI Insight. 2023 Oct 23;8(20):e164990. doi: 10.1172/jci.insight.164990 (PMC10619491; doi:10.1172/jci.insight.164990)
Supplement: Supplemental data [file jciinsight-8-164990-s025.pdf]

SUPPLEMENTARY INFORMATION

SET7/9-mediated methylation affects oncogenic functions of histone demethylase JMJD2A

Ruicai Gu, Tae-Dong Kim, Hoogeun Song, Yuan Sui, Sook Shin, Sangphil Oh, Ralf Janknecht

CONTENTS

Supplementary Tables 1 and 2

Supplementary Figures S1-S10

Supplementary References

Antibody	Vendor	Use
mouse monoclonal anti-Flag (M2)	Sigma-Aldrich F1804	IP, W
mouse monoclonal anti-Myc (9E10)	Sigma-Aldrich M4439	W
rabbit polyclonal anti-JMJD2A	Bethyl A300-861A	W
rabbit monoclonal anti-JMJD2A	Cell Signaling #3393	IP
rabbit IgG	Santa Cruz Biotechnology sc-2027	IP
rabbit polyclonal anti-p53-K372me$_1$	Abcam ab16033	W
rabbit polyclonal anti-H3K9me$_1$	Upstate/Sigma-Aldrich 07-450	W
rabbit polyclonal anti-SET7/9	Upstate/Sigma-Aldrich 07-314	W, IHC
rabbit polyclonal anti-androgen receptor	Upstate/Sigma-Aldrich 06-680	W
rabbit polyclonal anti-p53	Cell Signaling #9282	W
rabbit polyclonal anti-ETV1	#959 (homemade; Oh *et al* [1])	IP, W
rabbit polyclonal anti-MMP1	ThermoFisher Scientific-Invitrogen PA5-27210	W, IHC
rabbit polyclonal anti-NPM3	Novus Biologicals NBP1-90999	W, IHC
rabbit polyclonal anti-Actin	Sigma-Aldrich A2066	W
goat polyclonal anti-GAPDH	GenScript A00191-40	W

Supplementary Table 1. List of antibodies used for Western blotting (W), immunoprecipitation (IP) and/or immunohistochemistry (IHC).

Gene	NCBI Entrance	Direction	Sequence (5'→3')
MMP1 (Matrix metallopeptidase 1)	NM_002421.4	Forward<br>Reverse	TGTGGTGTCTCACAGCTTCC<br>CTGGCGTGTAATTTTCAATCCTGT
MMP14 (Matrix metallopeptidase 14)	NM_004995.4	Forward<br>Reverse	CCAGCAACTTTATGGGGGTGA<br>CAGGGACGCCTCATCAAACA
EPCAM (Epithelial cell adhesion molecule)	NM_002354.3	Forward<br>Reverse	GGCTGCCAAATGTTTGGTGAT<br>TCTGAAGTGCAGTCCGCAA
ITGB4 (Integrin subunit beta 4)	XM_006721868.4	Forward<br>Reverse	CTCCGATGACACTGAGCACC<br>CCCTTTCATCTGAGCCCGAC
PLAU (Plasminogen activator, urokinase)	NM_001145031.3	Forward<br>Reverse	GTCCTCCGGATTCCATCCAC<br>AGACAGTCACAGTTCGCCTG
NPM3 (Nucleophosmin/ nucleoplasmin 3)	NM_006993.3	Forward<br>Reverse	TTTGAGTCAGGAGAGCCGAAC<br>CATCCAGACTGAGCATGGGTT
FBLN1 (Fibulin 1)	NM_006486.3	Forward<br>Reverse	TCATCAGAAGGACTGCTCGC<br>CAATGGCAGCACCTCTTCAC
RNASEL (Ribonuclease L)	NM_021133.4	Forward<br>Reverse	GGACTTGGGAGAGCCGCTA<br>TCACCCACAGTGTTCTGGTAGA
GAPDH (Glyceraldehyde-3-phosphate dehydrogenase)	NM_002046.7	Forward<br>Reverse	GAGCCACATCGCTCAGACACC<br>TGACAAGCTTCCCGTTCTCAGC

Supplementary Table 2. Nucleotide sequences of the primers utilized for qRT-PCR.

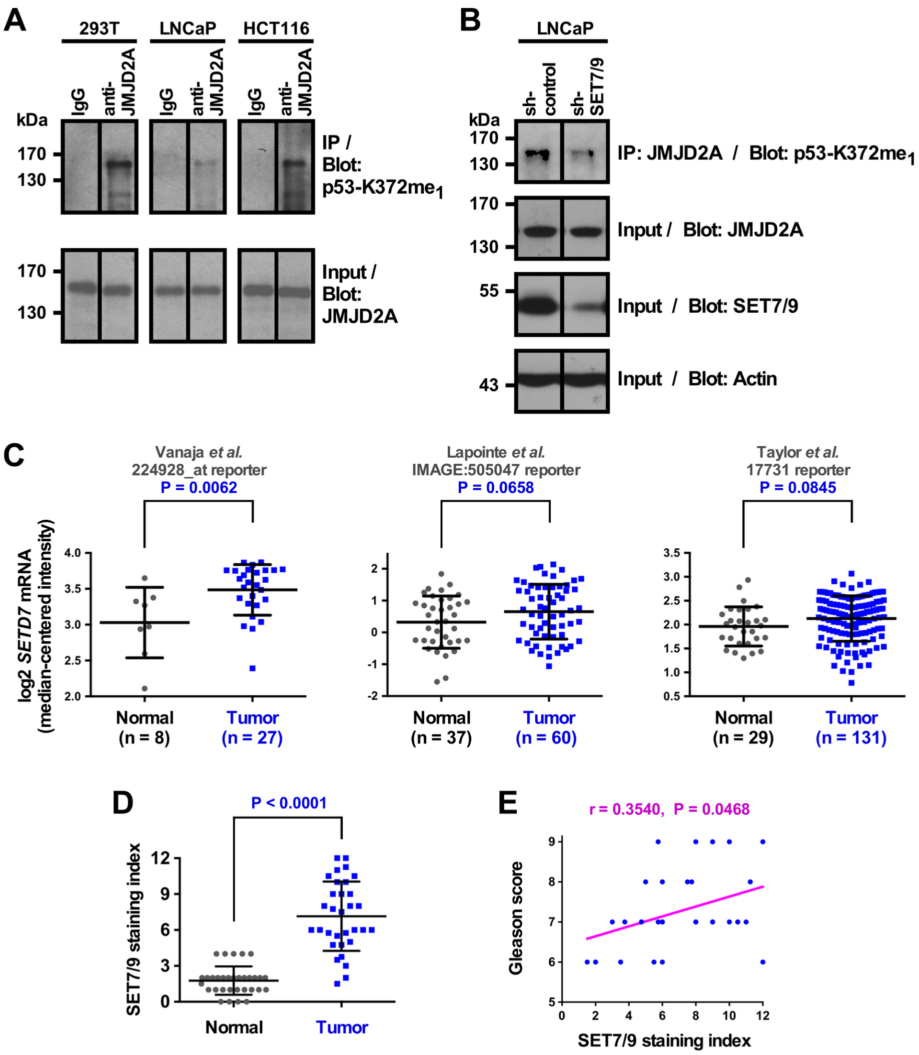

Supplementary Figure S1. (**A**) Methylation of JMJD2A in 293T, LNCaP and HCT116 cells. Lanes were run on the same gel but were noncontiguous. (**B**) Downregulation of SET7/9 (shRNA #1) leads to reduced JMJD2A methylation in LNCaP cells. Lanes were run on the same gel but were noncontiguous. (**C**) *SET7/9* (*SETD7*) mRNA levels in normal and cancerous prostate tissue. Microarray data were derived from Vanaja *et al* [2], Lapointe *et al* [3] or Taylor *et al* [4]. Means with standard deviations are shown; unpaired, two-tailed t-test. Statistical significance ($P<0.05$) for higher expression in tumors was noted in the Vanaja *et al* microarray data set, while a trend ($0.05 \leq P < 0.1$) for higher expression was present in the other two microarray data sets. (**D**) Immunohistochemical staining of SET7/9 was examined in 32 matching normal (1 core) and cancerous (mean of 2 cores) prostate specimens (AccuMax A302IV tissue microarray) and graded on a scale of 0-12; average of cytoplasmic and nuclear staining was calculated. Rabbit polyclonal SET7/9 antibodies (Upstate/Sigma-Aldrich 07-314, 1:400 dilution) were utilized for staining. Means with standard deviations are shown; paired, two-tailed t-test. (**E**) Correlation (Spearman) of the SET7/9 staining index with the Gleason score in the 32 prostate carcinomas analyzed in panel D. Regression line is shown in magenta color.

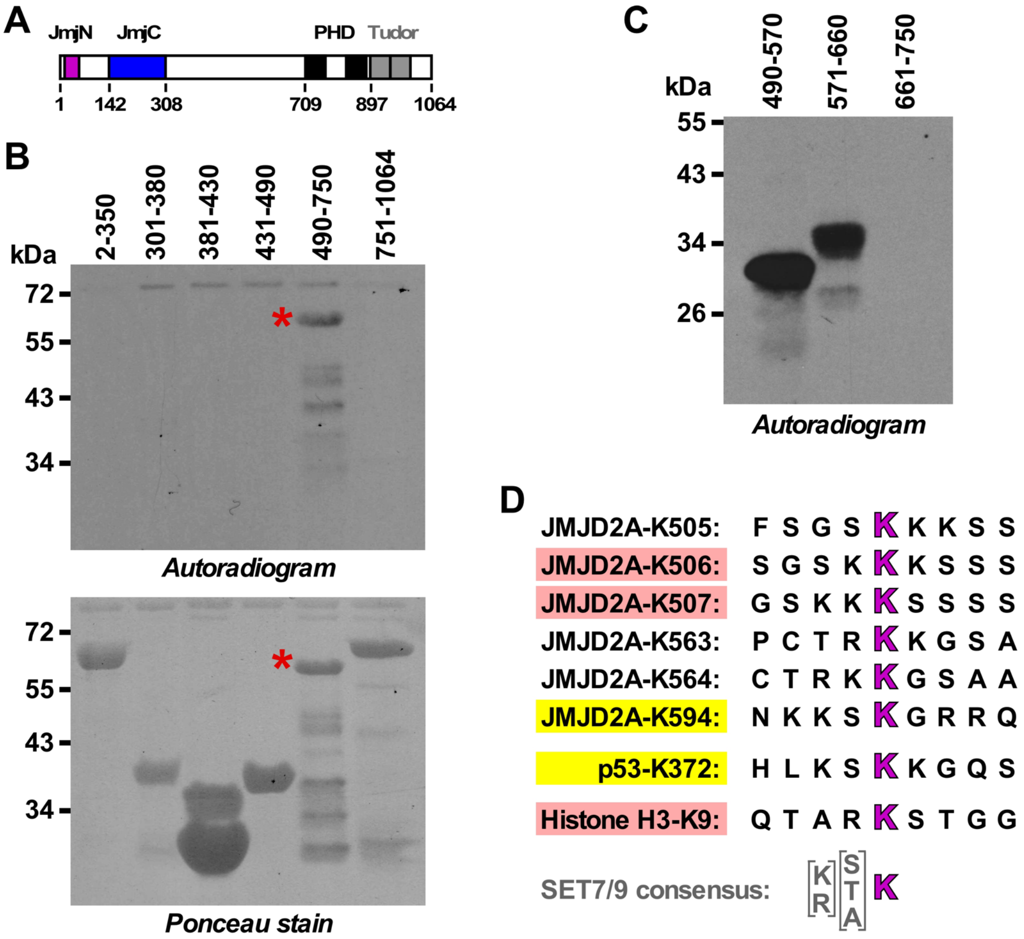

Supplementary Figure S2. Identification of methylated regions within JMJD2A. (**A**) Sketch of human JMJD2A displaying the catalytic JmjC domain, the double PHD and Tudor domains, and the N-terminal JmjN domain that is also required for catalytic activity. (**B**) SET7/9-mediated *in vitro* methylation of fusions between GST and indicated JMJD2A amino acids; S-[methyl-^{3}H] adenosyl-*L*-methionine (^{3}H-SAM) served as a methyl-donor. (**C**) Likewise, SET7/9-mediated *in vitro* methylation of comparable amounts of indicated GST-JMJD2A fusion proteins covering three different portions of JMJD2A amino acids 490-750. (**D**) Alignment of indicated methylation sites in JMJD2A, p53 and histone H3. Methylated sites recognized by the utilized p53-K372me$_1$ and H3K9me$_1$ antibodies are marked in yellow and beige color, respectively. The SET7/9 consensus methylation sequence is according to Couture *et al* [5].

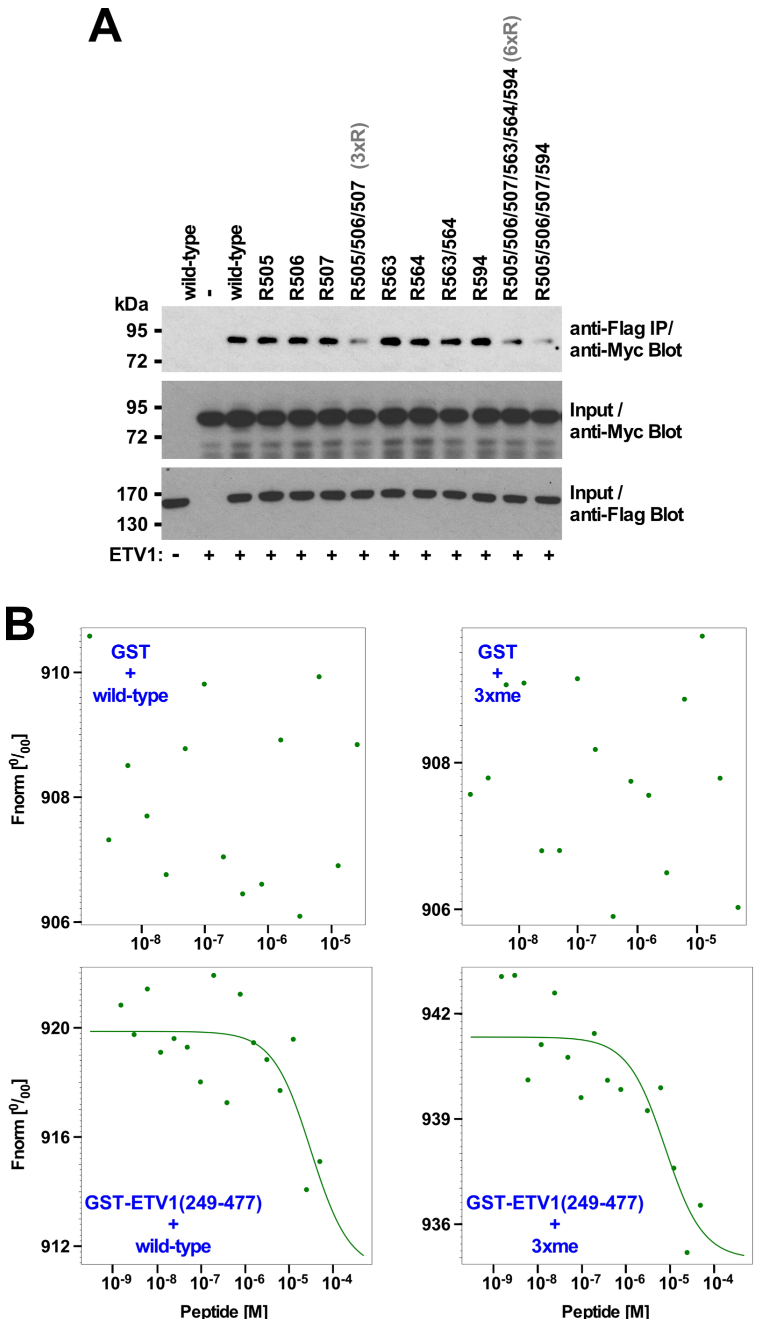

Supplementary Figure S3. (**A**) Coimmunoprecipitation of ETV1 with various JMJD2A methylation site mutants. Flag-tagged JMJD2A proteins (wild-type or indicated K→R mutants) were coexpressed with 6Myc-tagged ETV1 in 293T cells and immunoprecipitation (IP) was done with anti-Flag antibodies, followed by anti-Myc Western blotting. Bottom two panels show input levels for ETV1 and JMJD2A. (**B**) Examples of microscale thermophoresis experiments. No binding of wild-type or 3xme peptide was observed with the GST moiety (top), but binding occurred with the GST-ETV1(249-477) fusion protein (bottom).

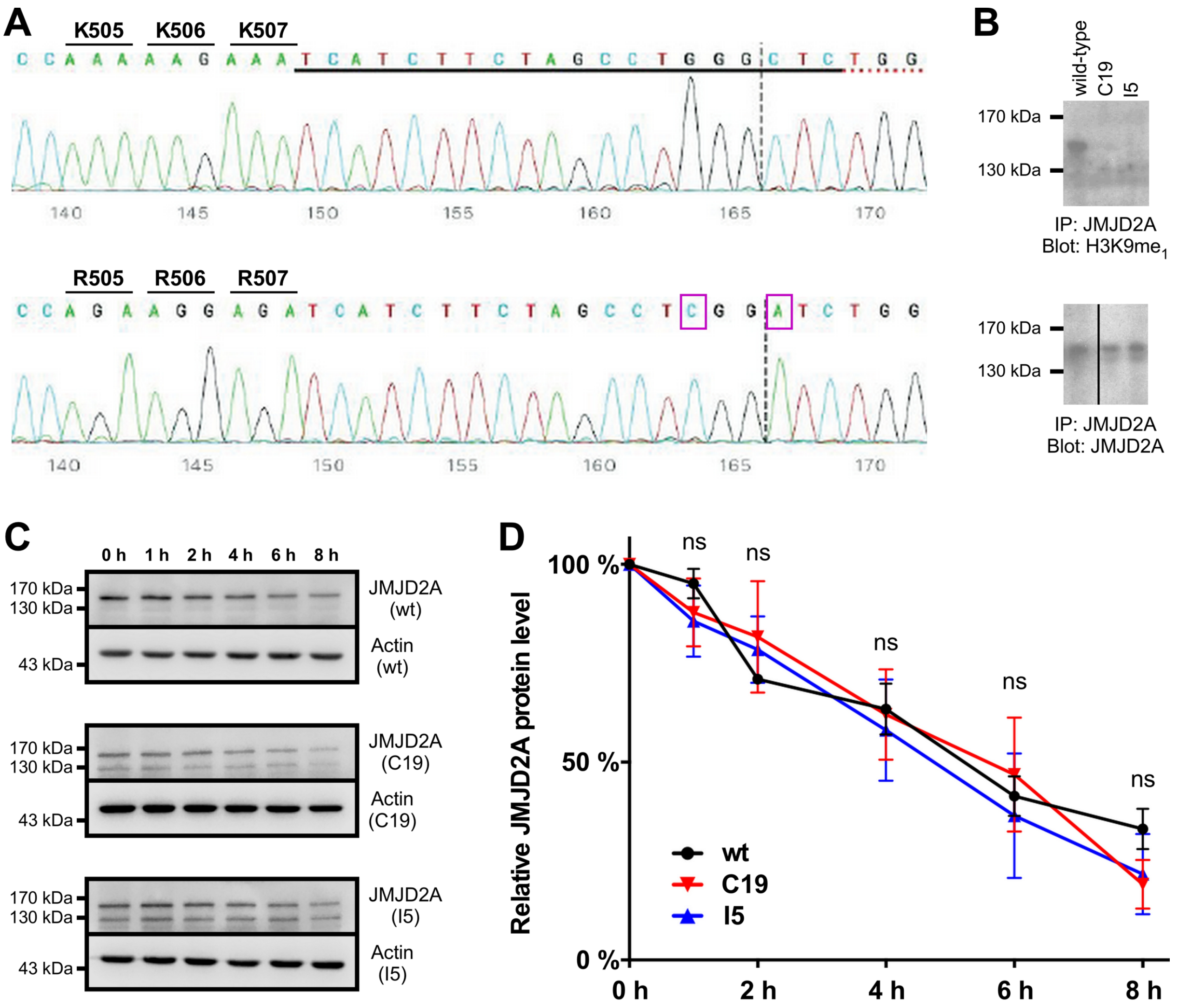

Supplementary Figure S4. 3xR knockin DU145 cells. (**A**) Homozygous gene knockin in DU145 cells utilizing CRISPR/Cas9 was done by Synthego (Redwood City, CA). Shown is the validation of DU145 JMJD2A-3xR mutant cells by Sanger sequencing of genomic DNA from wild-type cells (top) and one of the R505/506/507-mutated cell lines (3xR; bottom). One sgRNA sequence utilized in creating the knockin cells is underlined. Two silent mutations introduced to prevent re-cutting by Cas9 are boxed in magenta color. (**B**) Immunoprecipitation (IP) with anti-JMJD2A antibodies followed by blotting with anti-$H3K9me_1$ antibodies (which recognize K506/K507 methylation; see Fig. 2D) or anti-JMJD2A antibodies. Lanes on the bottom were run on the same gel but were noncontiguous. (**C**) Cells were treated for indicated time periods with 50 µg/ml cycloheximide. Shown are representative Western blots. (**D**) Corresponding quantitation of JMJD2A protein levels after normalization to actin levels. Shown are averages with standard deviation (n=3); two-way ANOVA (Tukey's multiple comparisons test); ns, not significant.

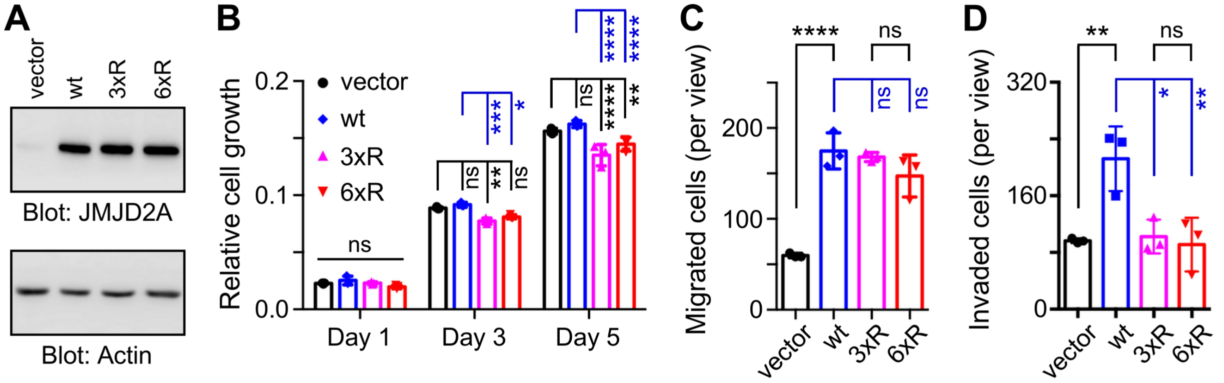

Supplementary Figure S5. Mutation of methylation sites compromises oncogenic activities of JMJD2A in human LNCaP prostate cancer cells. (**A**) Overexpression of wild-type (wt), 3xR (R505/506/507) or 6xR (R505/506/507/563/564/594) JMJD2A contrasted to the retroviral pQCXIH empty vector control. Comparable levels of recombinant JMJD2A proteins were observed by Western blotting. (**B**) Corresponding cell growth assay. Shown are averages with standard deviation (n=3); two-way ANOVA (Tukey's multiple comparisons test). (**C**) Migration and (**D**) invasion assays. Shown are averages with standard deviation (n=3); one-way ANOVA (Tukey's multiple comparisons test). ns, not significant. *, $P<0.05$. **, $P<0.01$. ***, $P<0.001$. ****, $P<0.0001$.

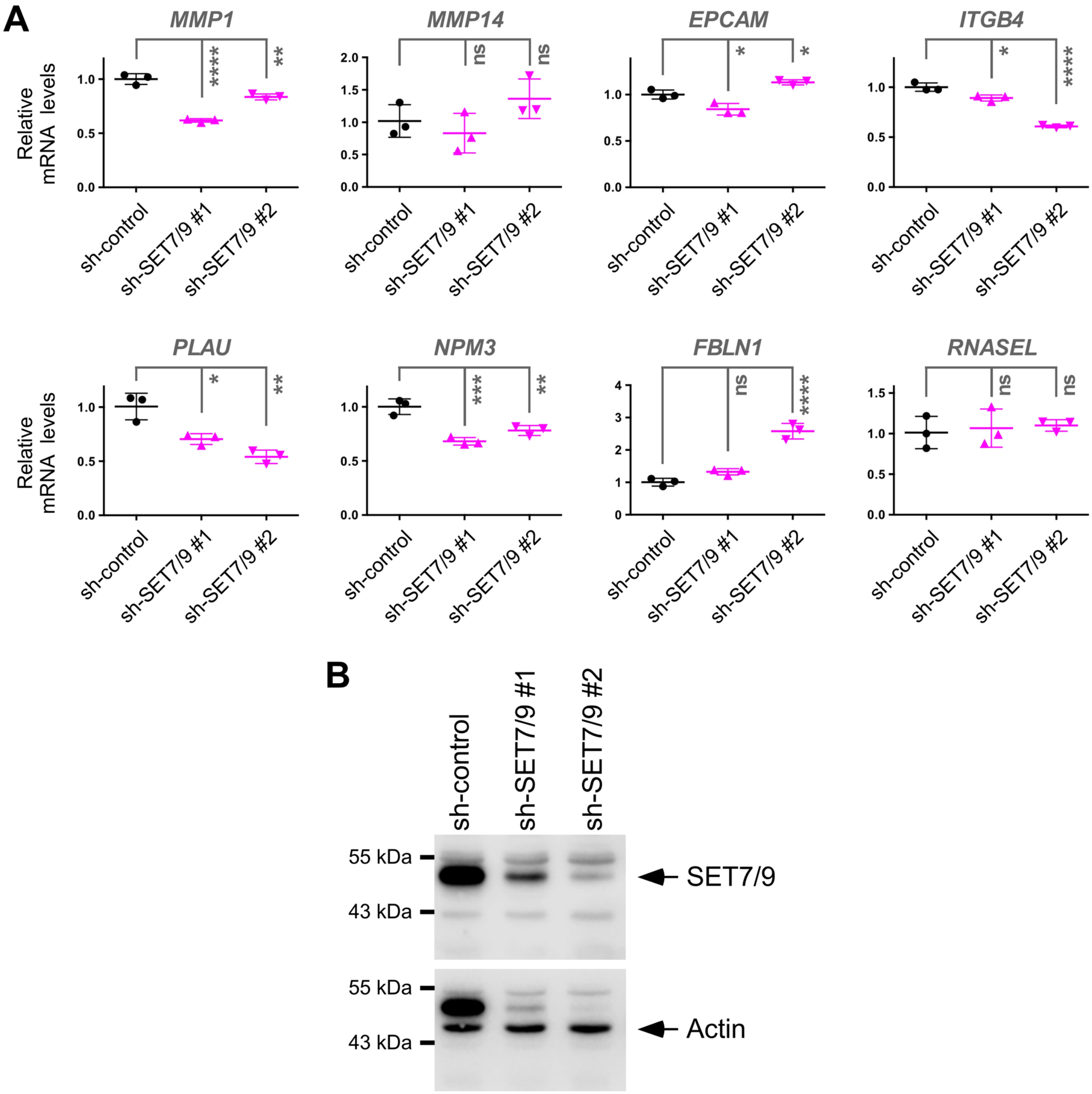

Supplementary Figure S6. Effects of SET7/9 downregulation on gene expression. (**A**) qRT-PCR with RNA isolated from DU145 cells that were infected with retrovirus expressing sh-control or two different SET7/9 shRNAs. Shown are relative mRNA levels (normalized to *GAPDH*). One-way ANOVA (Tukey's multiple comparisons test; n=3); ns, not significant; *, P<0.05; **, P<0.01; ***, P<0.001; ****, P<0.0001. (**B**) Corresponding Western blots for SET7/9 and Actin (loading control). Please note that the SET7/9 blot was re-probed (without prior stripping) with anti-Actin antibodies, which is why SET7/9 signals are also visible in the bottom blot.

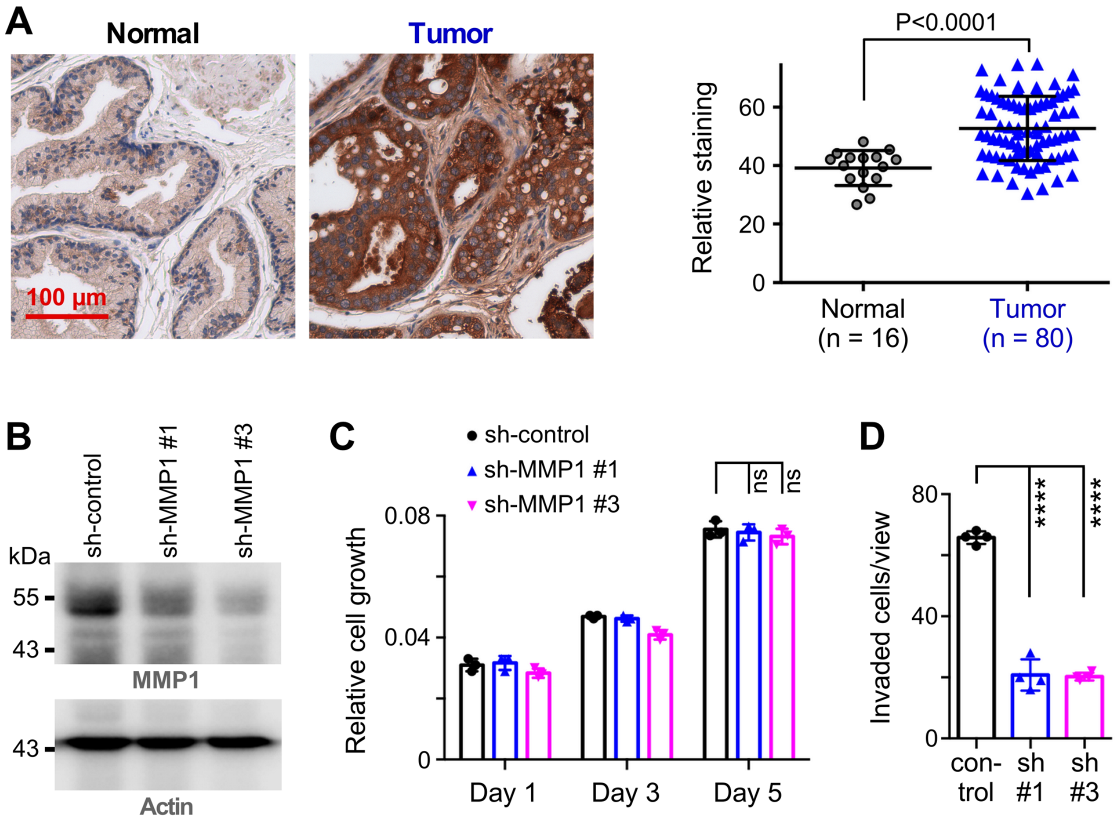

Supplementary Figure S7. MMP1 overexpression in prostate cancer and its role in invasion. (**A**) Example of MMP1 immunoreactivity in normal and cancerous prostate tissue and quantitation (unpaired, two-tailed t-test). A US Biomax PR1921c prostate cancer tissue microarray was stained with rabbit polyclonal anti-MMP1 antibodies (ThermoFisher Scientific-Invitrogen PA5-27210, 1:100 dilution). (**B**) Western blots showing downregulation of MMP1 with two different shRNAs in DU145 cells. The shRNAs were cloned into a retroviral vector (pSIREN-RetroQ) and targeted either 5'-GGUGGACCAACAAUUUCAG-3' (sh-MMP1 #1) or 5'-GAGCAAGAUGUGGACUUAG-3' (sh-MMP1 #3). (**C**) DU145 cell growth (n=3) and (**D**) invasion (n=4) were assessed after MMP1 downregulation. Shown are averages with standard deviation; two-way (panel C) or one-way (panel D) ANOVA (Tukey's multiple comparisons test). ns, not significant; ****, P<0.0001.

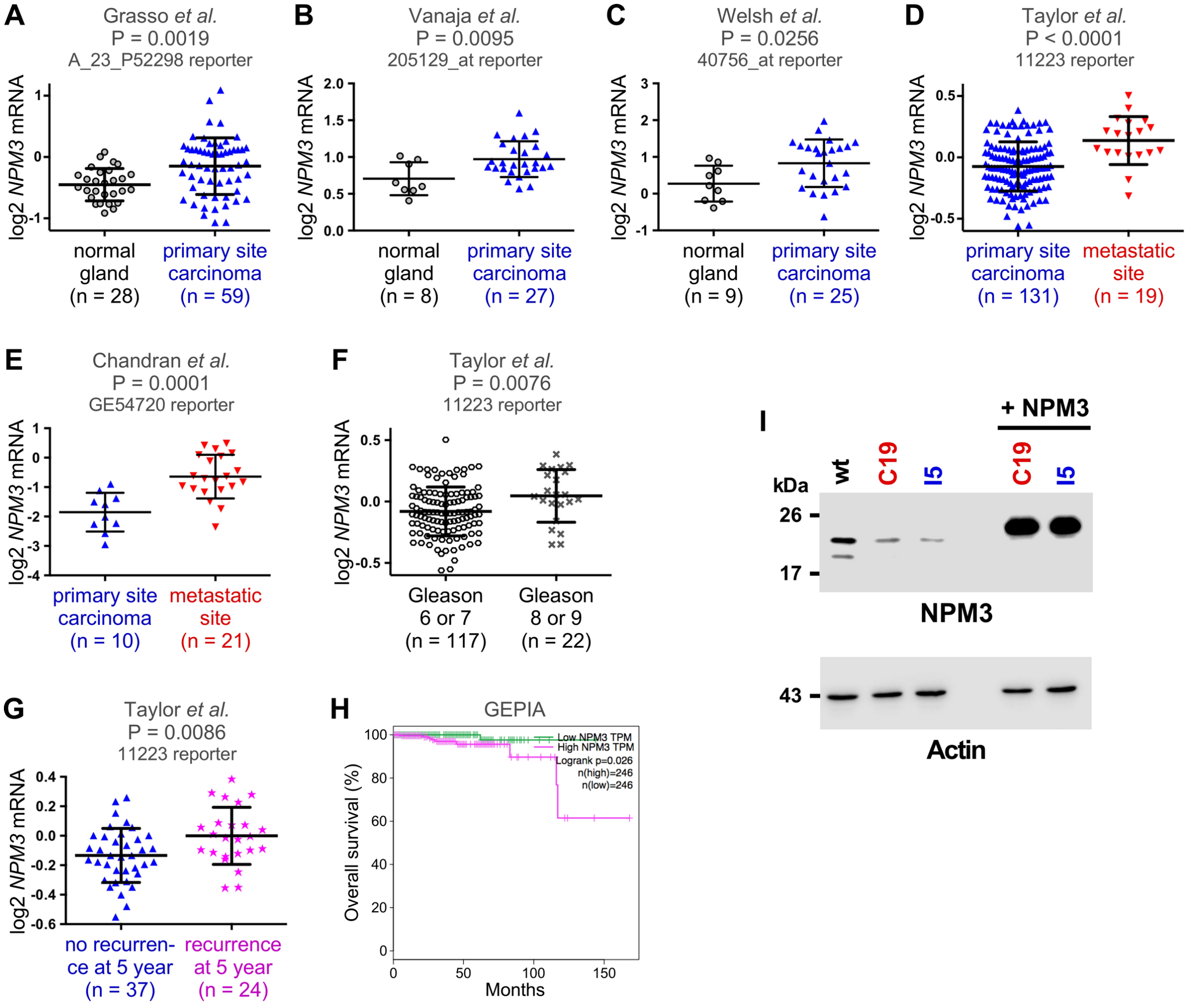

Supplementary Figure S8. *NPM3* overexpression in prostate cancer. (**A-E**) *NPM3* mRNA levels in normal prostate glands, in primary tumors or at metastatic sites. Microarray data were derived from Grasso *et al* [6], Vanaja *et al* [2], Welsh *et al* [7], Taylor *et al* [4] or Chandran *et al* [8]. Means with standard deviations are shown; unpaired, two-tailed t-test. (**F, G**) *NPM3* levels are higher in Gleason score 8/9 *versus* 6/7 tumors and also in tumors from patients experiencing recurrence of the disease 5 years after diagnosis. Microarray data were derived from Taylor *et al* [4]. Means with standard deviations are shown; unpaired, two-tailed t-test. (**H**) Kaplan-Meier survival analysis of 492 prostate adenocarcinomas in the TCGA database performed with the GEPIA (Gene Expression Profiling Interactive Analysis) webtool. High (magenta) or low (green) *NPM3* expression group each encompassed 246 patients. P = 0.026 (log-rank test). (**I**) Expression of endogenous or ectopic NPM3 in indicated DU145 cells. Shown are anti-NPM3 and anti-Actin Western blots. Due to an N-terminal Flag-tag, ectopic NPM3 has a slightly larger apparent molecular weight compared to endogenous NPM3.

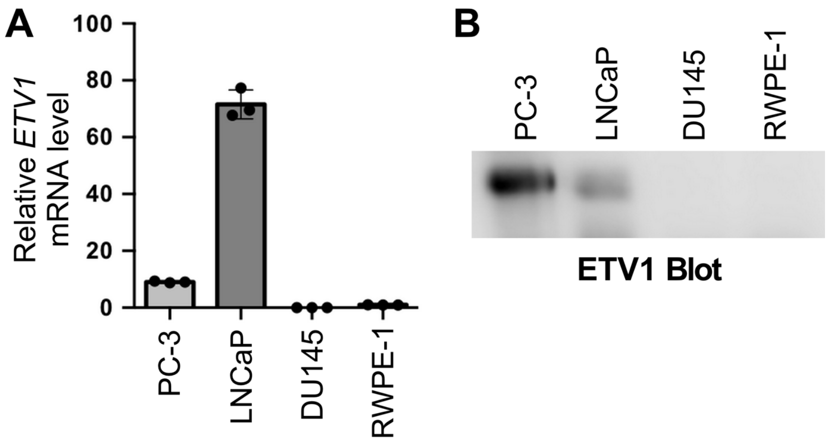

Supplementary Figure S9. Lack of ETV1 expression in DU145 prostate cancer cells. (**A**) *ETV1* mRNA levels were determined by qRT-PCR (normalized to *GAPDH*; n=3) as described (Oh *et al* [1]). While there was noticeable *ETV1* mRNA expression in PC-3 and LNCaP prostate cancer cells, there was little to none in DU145 prostate cancer cells and RWPE-1 prostate epithelial cells. (**B**) Immunoprecipitation of ETV1 protein followed by anti-ETV1 Western blotting. The utilized anti-ETV1 antibody was our homemade #959 one (described in Oh *et al* [1]). One ~95% confluent 10-cm dish of cultured cells was used for each immunoprecipitation.

```
                                                 505 506 507
JMJD2A  VKLEEEDEEEEQAAAALDLSVNPASVGGRLVFSGSKKKSSSSLGSGSSRD  519
JMJD2B  PVLGPGPAAMEESPLPAPLNVVPPEVPSEELEAKPRPIIPMLYVVPRPGK  537
JMJD2C  YAYRSVPSISSEADDSIPLSSG---------YEKPEKSDPSELSWPKSPE  512

                                                     563 564
JMJD2A  SISSDSETSEPLSCRAQGQTGVLTVHSYAKGDGRVTVGEPCTRKKGSAAR  569
JMJD2B  AAFNQEHVSCQQAFEHFAQKG--PTWKEPVSPMELTGPEDGAASSG-AGR  584
JMJD2C  SCSSVAESNGVLTEGEESD-----VESHGNG---LEPGEIPAVPSG-ERN  553

                                594
JMJD2A  SFSERELAEVADEYMFSLEENKKSKGRRQPLSKLPRHHP--LVLQECVSD  617
JMJD2B  METKARAGEGQAPSTFSKLKMEIKKSRRHPLGRPPTRSPLSVVKQEASSD  634
JMJD2C  SFKVPSIAEG---------ENKTSKSWRHPLSRPPARSPMTLVKQQAPSD  594
```

Supplementary Figure S10. Lack of conservation of SET7/9 methylation sites amongst the JMJD2A-C proteins. Shown is a partial amino acid alignment of JMJD2A (NCBI accession NP_055478.2) with JMJD2B (NCBI accession NP_055830.1) and JMJD2C (NCBI accession NP_055876.2). Identified methylation sites in JMJD2A are highlighted in grey, and conserved amino acids in yellow. Although JMJD2B and JMJD2C have lysine residues homologous to JMJD2A-K594 (K609 and K569, respectively), they – in contrast to K594 of JMJD2A – do not match to the SET7/9 consensus methylation sequence, (K/R)(S/T/A)**K**, that was reported by Couture *et al* [5]; further, our unpublished data indicate that K609 in JMJD2B does not become methylated by SET7/9, but we do not have any corresponding data for K569 of JMJD2C.

Supplementary References

1. Oh S, Shin S, Lightfoot SA, Janknecht R (2013) 14-3-3 proteins modulate the ETS transcription factor ETV1 in prostate cancer. *Cancer Res.* **73**, 5110-5119.
2. Vanaja DK, Cheville JC, Iturria SJ, Young CY (2003) Transcriptional silencing of zinc finger protein 185 identified by expression profiling is associated with prostate cancer progression. *Cancer Res.* **63**, 3877-3882.
3. Lapointe J, Li C, Higgins JP, van de Rijn M, Bair E, Montgomery K, Ferrari M, Egevad L, Rayford W, Bergerheim U, Ekman P, DeMarzo AM, Tibshirani R, Botstein D, Brown PO, Brooks JD, Pollack JR (2004) Gene expression profiling identifies clinically relevant subtypes of prostate cancer. *Proc. Natl. Acad. Sci. USA* **101**, 811-816.
4. Taylor BS, Schultz N, Hieronymus H, Gopalan A, Xiao Y, Carver BS, Arora VK, Kaushik P, Cerami E, Reva B, Antipin Y, Mitsiades N, Landers T, Dolgalev I, Major JE, Wilson M, Socci ND, Lash AE, Heguy A, Eastham JA, Scher HI, Reuter VE, Scardino PT, Sander C, Sawyers CL, Gerald WL (2010) Integrative genomic profiling of human prostate cancer. *Cancer Cell* **18**, 11-22.
5. Couture JF, Collazo E, Hauk G, Trievel RC (2006) Structural basis for the methylation site specificity of SET7/9. *Nat. Struct. Mol. Biol.* **13**, 140-146.
6. Grasso CS, Wu YM, Robinson DR, Cao X, Dhanasekaran SM, Khan AP, Quist MJ, Jing X, Lonigro RJ, Brenner JC, Asangani IA, Ateeq B, Chun SY, Siddiqui J, Sam L, Anstett M, Mehra R, Prensner JR, Palanisamy N, Ryslik GA, Vandin F, Raphael BJ, Kunju LP, Rhodes DR, Pienta KJ, Chinnaiyan AM, Tomlins SA (2012) The mutational landscape of lethal castration-resistant prostate cancer. *Nature* **487**, 239-243.
7. Welsh JB, Sapinoso LM, Su AI, Kern SG, Wang-Rodriguez J, Moskaluk CA, Frierson HF, Jr., Hampton GM (2001) Analysis of gene expression identifies candidate markers and pharmacological targets in prostate cancer. *Cancer Res.* **61**, 5974-5978.
8. Chandran UR, Ma C, Dhir R, Bisceglia M, Lyons-Weiler M, Liang W, Michalopoulos G, Becich M, Monzon FA (2007) Gene expression profiles of prostate cancer reveal involvement of multiple molecular pathways in the metastatic process. *BMC Cancer* **7**, 64.
